# Non-union after bisphosphonate-associated atypical femoral fractures: a review of the current literature

**DOI:** 10.1007/s00590-026-04677-2

**Published:** 2026-02-18

**Authors:** Jack Dowling, Samuel Marsh, Jamie O’Grady

**Affiliations:** Royal Perth Hospital, Wellington Street, Perth, Western Australia Australia

**Keywords:** Atypical femoral fractures, Bisphosphonates, Non-union, Management options, Revision

## Abstract

**Background:**

This article explores the mechanism of action of atypical femoral fractures (AFF), specifically focussing on osteoporosis, management challenges, and the implications for clinical practice. Non-union of AFF can result in severe pain, deformity and restricted mobility—all of which will have a detrimental impact on the patients’ quality of life.

**Purpose:**

This review consolidates the current literature regarding non-union after bisphosphonate-associated atypical femoral fractures, providing a revision tool for higher examinations.

**Method/findings:**

An extensive search strategy was conducted for relevant studies up to April 2025 using several online databases including Cochrane Library, Embase, National Library of Medicine and PubMed.

**Conclusion:**

Although this topic is prominent among surgical colleagues and in higher level examinations, we identified that persistent gaps in knowledge exist in this area. Our review compiles the available knowledge as a revision tool for our colleagues.

## Introduction

Bisphosphonate agents play a key role in the medical management of numerous disease processes; namely Paget’s disease of bone, malignant metastasis to bone, multiple myeloma, hypercalcemia, and osteoporosis [1].

This article explores the mechanism of action of bisphosphonates, particularly in osteoporosis, their association with atypical femoral fractures (AFF), management challenges, and clinical implications.

AFFs are particularly associated with non-union—which is defined by the Food and Drug Administration as a fracture that persists for a minimum of nine months without signs of healing for at least three months [2].

AFFs are a significant issue across orthopaedic patients. The absolute relative risk of AFF among women is 110 fractures per 100,000 person-years of use [3].

Wysowki et al. 2013. reviewed nationwide prescriptions for oral bisphosphonates dispensed from retail pharmacies across the United States of America. They found that prescribing peaked with 31 million prescriptions of bisphosphonates in 2007 [4].

## Background

### Bisphosphonates mechanism of action

Bisphosphonates are traditionally considered to all share the same mechanism of action, but are structurally diverse; categorized as either nitrogen or non-nitrogen containing agents.

Bisphosphonates are pyrophosphate analogues, which bind strongly to the bone mineral hydroxyapatite. This occurs where the mineral is exposed to surrounding fluids—acting at sites of formation and resorption in periosteal and cortical bone [5].

Bisphosphonates are anti-resorptive agents that can be considered to act across three levels—molecular, cellular and tissue [6].

Nitrogen-containing bisphosphonates, such as Zolendronic acid, are potent anti-resorptive agents via additional inhibition of the mevalonate molecular pathways [7]. These agents inhibit farnesyl pyrophosphate synthase - an essential element of intracellular osteoclast function, and thus induce osteoclast apoptosis [8].

Bisphosphonates act at a cellular level via direct and indirect action upon the osteoclast. Reduced resorption occurs with inhibition of progenitor differentiation, osteoclast recruitment, activity on the bone surface, shortened lifespan and alteration of the bone mineral itself, reducing the rate of dissolution [6].

Bisphosphonate action is seen at a tissue level with decreased resorption and increased mineral content [6]. Coupling refers to bisphosphonate action of decreased bone turnover by decreased resorption. This results in a positive focal bone balance and relatively rapid gain in bone mass [6].

Bisphosphonate action creates brittle cortical bone at risk of fracture. Bisphosphonate inhibition of bone remodelling allows microdamage accumulation and bone becoming increasingly brittle [9, 10]. Boskey et al. 2013, a systematic review compared several bisphosphonates with respect to bone mineral density and bone composition measured via FTIR spectroscopy. They showed that all bisphosphonates, especially alendronate, reduced tissue heterogeneity and thus increased fracture risk [11].

Biochemical factors, such as femoral bone geometry and mechanical stresses, influence location of initial sites of failure. Femoral bowing creates tensile force on the lateral cortex and it is the lower limb geometry that influences the site of maximal tensile stress. Saita et al. 2014, showed that patients with larger femororbital angle (FTA) (more varus) had increased propensity to diaphyseal lesions (mid-distal); while patients with smaller FTA’s (more valgus) tended towards subtrochanteric lesions (proximal) [12]. Oh et al. 2014 used CT imaging to evaluate bone morphology to show a marked concentration of tensile stress along the anterolateral surface of a bowed AFF [13].

Periosteal stress reaction, specifically cortical beaking, is characteristic of early AFF. Beaking occurs at the site of this maximal tensile stress, initially fracturing of the lateral femoral cortex. This fracture then continues through to the medial cortex [14]. These fractures can then become oblique, as described by Goh et al. 2008 [15].

### Atypical femoral fractures

Atypical femoral fractures were defined by the American Society for Bone and Mineral Research in their Task Force completed in 2013 [16].

To satisfy the case definition of AFF, the fracture must be located along the femoral diaphysis from just distal to the lesser trochanter to just proximal to the supracondylar flare.

In addition, at least four of five Major Features must be present. None of the Minor Features are required but have sometimes been associated with these fractures.

Major features:

1. The fracture is associated with minimal or no trauma, as in a fall from a standing height or less.
2. The fracture line originates at the lateral cortex and is substantially transverse in its orientation, although it may become oblique as it progresses medially across the femur.
3. Complete fractures extend through both cortices and may be associated with a medial spike; incomplete fractures involve only the lateral cortex.
4. The fracture is noncomminuted or minimally comminuted.
5. Localized periosteal or endosteal thickening of the lateral cortex is present at the fracture site (“beaking” or “flaring”).

Minor features:

1. Generalized increase in cortical thickness of the femoral diaphyses.
2. Unilateral or bilateral prodromal symptoms such as dull or aching pain in the groin or thigh.
3. Bilateral incomplete or complete femoral diaphysis fractures.
4. Delayed fracture healing.

Gedmintas et al. 2013, showed, in a meta-analysis of 11 papers, that bisphosphonate exposure was associated with an increased risk of subtrochanteric, femoral shaft, and AFF, with adjusted relative risk of 1.70 [17]. Dennis et al. 2020, showed in an extensive study involving 196,129 women over 50 years old, on bisphosphonate therapy, for 10 years, resulted in 277 atypical femoral fractures [18]. This conveys a low absolute risk and overall favourable risk–benefit analysis.

A systematic review conducted by Molvik et al. explored where fractures were most likely to occur in patients established on bisphosphonate therapy for a mean of 5.4 years [19]. Out of 3542 patients reviewed, 66.8% had femoral fractures, 25.1% had humeral fractures, 6.95% had radial fractures, with small numbers of rib, metatarsal, fibular, ankle and pelvic fractures reported [19]. Shane et al. found a 28% rate of bilateral fractures in patients with bisphosphonate-associated femoral fractures [16].

The literature would suggest that AFFs are the more common of femoral fractures in patients on bisphosphonate therapy [20, 21].

Shkolnikova et al. performed a single-centre, 5-year, retrospective review of all diaphyseal and subtrochanteric fractures in Melbourne. 30.3% of these fractures were atypical in nature, and of those AFFs, 90% were established on bisphosphonate therapy prior to the fracture occurring [21]. A single-centre retrospective review conducted by Girgis et al. showed a similar rate of 85% of AFFs occurring in patients established on bisphosphonate therapy [20]. Furthermore, the literature would suggest that the risk of AFF increases with time, in patients on bisphosphonate therapy, while the rate of neck of femur and intertrochanteric fractures does not [3, 18]. In a single-centre, retrospective, case–control study of all low-energy femoral fractures, 15 of the 41 subtrochanteric fractures (36.6%) and 9 of the 81 neck of femur and intertrochanteric fractures (11.1%) were in patients established on bisphosphonate therapy [22].

One retrospective review of 172 AFFs, having reviewed 5342 radiographs, found that the relative risk of AFF, among women, increased annually, with the relative risk at 4 years being 126.0, with an absolute risk of 11 fractures per 10,000 person-years of use [3].

### Implication of AFFs

Fracture healing is a complex process relying on cellular recruitment, proliferation, differentiation, extracellular matrix deposition and mineralisation. Non-union can result from the interaction between a number of key factors [23]:

- Patient related factors (genetics, systemic disorder, osteoporosis, age)
- Environmental factors (smoking, medications, alcohol)
- Injury characteristics (high energy, vascular injury, soft tissue involvement, contamination, localisation, fracture pattern)
- Treatment modality (vascular supply, soft tissue interposition, distracted gap reduction > 2 cm, malposition, infection) [23].

### Presentation and early recognition

Post-fracture bisphosphonate use was associated with approximately doubling of risk of non-union [24]—highlighting the need for early recognition in this cohort.

A common complaint of patients who go on to have AFFs associated with bisphosphonate therapy is prodromal pain in the ipsilateral thigh [16, 25–27]. A task force for the American society for Bone and Mineral research found that 70% of patients established on bisphosphonate therapy experienced prodromal pain in the ipsilateral thigh for weeks prior to diagnosis of an AFF [16]. A National screen in Sweden showed an 86% rate of the same ipsilateral thigh pain. This used a smaller patient cohort of 45 compared to 227 [25]. Single institution retrospective reviews showed similar rates of this prodromal pain [16, 25].

Prior to diagnosis of the fracture, some radiological changes have been found to be associated with bisphosphonate-related AFFs [22, 28, 29]. Radiological cortical beaking being the primary finding. Neviaser et al. conducted a retrospective review of AFFs [29]. Of the 70 patients identified in the review, 25 had been established on bisphosphonate therapy for a mean of 6.2 years. Of these 25, 19 of the fractures had evidence of thickened cortices with a cortical beak [28]. A single centre, 10-year, retrospective review of patients on established bisphosphonate therapy, had a similar rate of lateral cortical beaking (75% vs 76%) [28]. Lenart et al. having conducted a retrospective case–control study, found a similar rate of 66.67%, of bisphosphonate-associated AFFs, having this radiological pattern [22].

#### Rates of non-union amongst bisphosphonate associated AFFs

While delayed union is an ongoing issue in this patient cohort, with one systematic review demonstrating a mean time to union of femoral fractures of 38.2 weeks, compared to 19.08 weeks in control groups [19], we will be primarily focusing on non-union in bisphosphonate-associated fractures in this article.

Following a brief search on PubMed, with search criteria of ‘Bisphosphonate’ AND ‘Fracture’ AND ‘non-union’, 4 pieces of primary research were identified [21, 30–32], 3 of which were retrospective reviews of outcomes in patients with AFF, established on bisphosphonate therapy. Şahin et al. demonstrated a 21.05% of non-union in their retrospective review [31], with a retrospective cohort study conducted by Kayali et al. showing a similar rate of 22.7% [33]. Teo et al. and Shkolnikova et al. demonstrated lower non-union rates of 11.1% and 3.03% of patients established on bisphosphonate therapy, respectively [21, 32]. Solomon et al. conducted a case–control study demonstrating that there was no increase in the rate of non-union in humeral fracture, in patients established on bisphosphonate use prior to the fracture occurring [30].

2 literature reviews were identified in our search. These demonstrated the wide range of rates of non-union. Molvik et al. included 1275 patients, across 6 papers, with a mean non-union rate of 1.25% [19]. Comparatively Yue et al. found a mean non-union rate of 20%, across 14 papers, including 420 patients, however, these rates ranged from 2 to 63% [34].

All of these papers observing for non-union included patients who were established on bisphosphonate therapy for at least 5 years. Koh et al. showed in their systematic review that only a marginal difference between rates of non-union between groups treated with bisphosphonates for more than 5 years, compared to less than 5 years; 24% vs 20.4%, respectively [35].

### Treatment

There are many documented approaches to the treatment of non-union in the literature, with few specifically for the treatment of non-union in bisphosphonate associated fractures.

Treatment for non-union associated with bisphosphonate therapy can be divided into surgical and medical management. As described above, the most common site for bisphosphonate-associated fractures to occur is in the femur, followed by the humerus and then the radius [19]. The majority of primary research dedicated to bisphosphonate-associated non-union is also shown to pertain to femoral fractures, as above [21, 30–32]. Because of this, the strongest research for the treatment of bisphosphonate-associated non-union is for AFFs.

#### Medical approach to non-union of bisphosphonate-associated AFFs

Medical management is accessible as soon as non-union is established, and so should be initiated immediately. Medical management also does not prohibit a surgical approach, and so should be initiated even if the patient requires surgical management [16, 36].

#### Ceasing bisphosphonate therapy

The mainstay of medical management of bisphosphonate-associated non-union is to discontinue bisphosphonate therapy. Shane et al. [16] stated that in the case of a bony stress reaction, stress fracture, incomplete subtrochanteric, complete subtrochanteric and femoral shaft fractures, treatment with bisphosphonates should be ceased. Discontinuation of bisphosphonates allows normal resorption and remodelling to recommence. In a report conducted by Odvina et al. [37] 9 subjects were identified who had sustained spontaneous non-spinal fractures while on alendronate therapy. This study’s histomorphometric analysis of cancellous bone suggested that reduced bone turnover could lead to delayed fracture healing, potentially resulting in non-union. After stopping bisphosphonate therapy, 4 patients suffered from non-union, while the remaining 5 progressed to satisfactory bone healing.

#### Teriparatide

Shane et al. [16] go on to explain how teriparatide, a recombinant fragment of endogenous parathyroid hormone used in the treatment of osteoporosis [38] has also been shown to aid in bone healing after bisphosphonate-associated fractures. Teriparatide stimulates bone formation at the site of non-union by activating osteoblasts. There is also systemic increase in calcium levels via renal and intestinal reabsorption which is essential for fracture-callus mineralisation [39]. Evidence for use of teriparatide to aid healing after bisphosphonate-associated non-union has shown varied results. A systematic review by Gun-Il Im et al. [40] reviewed the effect of teriparatide on healing of AFFs. This review notes the limited research available on this topic, and is predominantly composed of case reports and retrospective studies. They note that treatment of non-union with teriparatide reduces union time and lowers the occurrence of delayed or non-union in surgically treated AFFs. Saleh et al. also show that treatment with teriparatide is most effective in stable fracture sites in both surgical and non-surgical cases [41]. In conclusion, teriparatide therapy is shown as an effective treatment for non-union in bisphosphonate-associated fractures and should be considered in patients who have not achieved union with conservative therapy [16].

Beyond these medical treatments, it is also recommended to consider non-specific treatment of non-union such as review of dietary calcium and vitamin D and provide supplementation where indicated [16, 42, 43].

#### Surgical approach to non-union of bisphosphonate-associated AFFs

There are different approaches to surgical management for non-union in bisphosphonate associated fractures. Initial management of bisphosphonate-associated fractures may include intramedullary nail fixation, as well as for incomplete fractures which are accompanied by pain. Prophylactic nail fixation should also be strongly considered in patients who fail conservative therapy (cessation of bisphosphonates and limited weight bearing through the affected bone) after 2–3 months [16].

If a patient suffers from non-union after these primary interventions, they may be offered further surgical management. Compression plating is a treatment option that shows success in both cases in the case series by Grady et al. [44]. Nagy et al. [36] performed a retrospective study of ten consecutive cases of revision surgery for non-union of bisphosphonate related subtrochanteric fractures. Their surgical approach achieved successful union in all cases by following these principles: resection of the fractured bone at the site of non-union, bone graft, valgus neck shaft alignment and rigid fixation using double plating.

#### Resection of non-union and bone graft

Resection of failed bone at the site of non-union should be performed in any case of non-union [45]. After resection, it is generally recommended to perform bone graft augmentation to fill gaps where bone has been resected and to maintain stability [46]. Autologous bone graft is advantageous over allograft in the setting of non-union as it is superior for osteoinduction, osteoconduction and osteogenicity of the graft. Autologous graft also has inherent immune compatibility with the patient and no risk of disease transmission [47, 48].

#### Valgus neck shaft alignment

A fracture that has been reduced in varus significant increases the risk for fracture non-union [49–51]. Valgisation in conjunction with anatomical reduction are essential for achieving fracture union [52]. The difference in healing times is highlighted in a retrospective study by Egol et al. [53] who found that anatomically reduced fractures healed 3.7 months faster than those fixed in a varus alignment.

#### Rigid fixation

Rigid fixation is one of the tenets of non-union repair [45]. Nagy et al. [36] approached fixation using a lateral dynamic compression screw (DCS) plate and an anterior locking compression plate (LCP) plate. The lateral DCS plate serves to stabilise the fracture in the coronal plane but does not provide sufficient stabilisation in the sagittal plane, hence the anterior LCP was applied to provide stability along this plane. This double plating compresses and rigidly immobilises the fracture site which promotes union [54]. This double plate fixation was enhanced by the ipsilateral iliac crest autologous bone graft as described above. However, a fixation that is too stiff, will risk failure of the construct itself or the bone around it.

This study by Nagy et al. [36] notes that if a bisphosphonate associated fracture requires surgical treatment, the treating doctor should be aware that time to union may be prolonged. In their study the average time to achieve union was 16 months.

Intramedullary nailing is the recommended surgical treatment for patients who have already received prophylactic surgical fixation and have subsequently suffered non-union. This ‘exchange nail’ should be at least 2 mm larger in diameter than the previously implanted nail, and should be fixed with the widest available locking screws proximally and distally. Principles for this surgery are similar to the compression plating technique described above, and include: resection of fibrous tissue, fresh reaming of femoral canal, circumferential pedalling of bone at the site of non-union to expose bleeding bone, and bone grafting. This technique described by Leighton et al. [55] also advises that if the exchange nail fails and non-union occurs a second time, nail removal and compression plating with the technique described above should be considered.

## Discussion

The literature as shown demonstrates that bisphosphonate therapy is associated with an increased risk of AFF. Ipsilateral thigh pain is an established prodromal feature of AFF [16, 25–27]. Clinicians should be aware of the same in their ongoing management of patients on bisphosphonate therapy. The research demonstrates that there are well-defined radiographic features that precede AFFs in patients on bisphosphonate therapy, in lateral cortical beaking [3, 13, 28]. Given this well-defined prodrome with radiographic evidence to support it, the literature suggests that clinicians may be able to identify at-risk patients before the AFF occurs.

Once the AFF does occur, there are wide ranging rates of non-union documented in the literature. The 2 systematic reviews, discussed above, showed a 20% vs 1.25% mean rate of non-union in AFFs associated with bisphosphonate therapy [19, 34]. Both of these studies had large population groups, and yet, the rates of non-union varied greatly, still. It is clear that further primary research is needed in order to clarify if there is a consistent rate of non-union of AFFs amongst patients established on bisphosphonate therapy.

While the identification of non-union in bisphosphonate-associated fractures is well reviewed in the literature, its treatment is not widely addressed. There is a requirement for further dedicated research that is specific to the treatment of non-union in the context of bisphosphonate use, rather than general treatment measures for normal non-union. Where dedicated research does exist, study sizes are small. Larger patient cohort samples are required to establish stronger evidence, and if shown to be beneficial could lead to improved patient outcomes.

The evidence for teriparatide use to aid healing after bisphosphonate-associated non-union is not well established. Gun-Il Im et al. [40] note the limited research available on this topic. Although their research method is impactful in its method of systematic review, they note that the primary research available is predominantly composed of case reports and retrospective studies. If cohort studies and randomised control trials were available, the role of teriparatide could be better defined. The use of teriparatide is shown as an effective treatment for non-union in bisphosphonate-associated fractures and should be considered in patients who have not achieved union with conservative therapy [16].

Beyond these treatments, there is scope to assess the effectiveness of other emerging treatments of non-union in this specific patient cohort i.e. those on bisphosphonate therapy. For example, Low Intensity Pulsed Ultrasound (LIPUS) is a new treatment option that accelerates healing at sites of non-union [56]. LIPUS serves as an alternative to revision surgery which many patients in this cohort require. LIPUS and other emerging treatments for non-union should be evaluated for effectiveness in bisphosphonate-associated non-union.

Surgical treatments of non-union in bisphosphonate-associated non-union also display limited evidence. While all cases reviewed by Grady et al. and Nagy et al. show compression plating as a successful treatment option, their research only reviews 2 and 10 patients respectively [36, 44]. Leighton et al. describes exchange nailing in patients who have suffered non-union after prophylactic nailing, but this paper only includes the case report of a single patient [55].

In short, there are established treatments available for patients on bisphosphonates who have suffered non-union after a fracture. However, these treatment methods require deeper research and analysis, so that other clinicians may provide timely and more effective management plans to ensure improved patient outcomes.

Previously, non-union in these cases was attributed to poor bone healing due to bisphosphonates alone, but patient factors also exist. Patients should be considered on an individualised basis. In terms of key aspects to management, we recommend a step-wise evidence-based approach to the issue. First, recognition of non-union followed by discontinuation of the offending medication. Second, a thorough blood screen including calcium, vitamin D and parathyroid hormone to correct any metabolic deficiencies that may compound the problem. Third, early use of the multi-disciplinary team using a patient-centred care model. Factors such as mobility status, compliance with weight-bearing status, nutritional status, social support, smoking, chronic illnesses and cognitive function will impact management. Fourth, radiological assessment to evaluate implant integrity and operative options, as well as screening of the contralateral femur. Fifth, intramedullary nailing focussing on optimal surgical technique with particular attention to anatomical reduction while minimising varus malreduction and fracture gap. Sixth, implementation of a bisphosphate holiday and consideration of additional pharmacological therapies – such as teriparatide. Seventh, close follow-up in the post-operative phase.

## Strengths and limitations

There were a number of limitations found during our review of the literature. The understanding of bisphosphonate action creating an environment for microdamage accumulation, is based off seminal work by Mashiba T. et al. and Li et al. (9, 10). Together, these papers have been cited more than 780 times. However, the mechanism by which this occurs has only been demonstrated in dog and rat models.

There is need for further research into established treatment options with use of globally recognised and standardised metrics. While teriparatide has been shown to be effective in management, there is limited evidence such as a prospective randomised control trials into its use. This will enable research into emerging treatment options with clinical implications.

## Conclusion

Atypical femoral fractures occur due to multiple pathogenic processes. They cause a number of challenges in management, particularly that of non-union. The article consolidates the current literature, which may serve as a valuable revision resource for higher orthopaedic examinations and help address gaps in knowledge.

## Data Availability

No datasets were generated or analysed during the current study.
